# Dapagliflozin reduces risk of heart failure rehospitalization in diabetic acute myocardial infarction patients: a propensity score-matched analysis

**DOI:** 10.1007/s00228-023-03495-3

**Published:** 2023-04-26

**Authors:** Lipeng Mao, Dabei Cai, Boyu Chi, Tingting Xiao, Ailin Zou, Yu Wang, Qianwen Chen, Qingqing Gu, Qingjie Wang, Yuan Ji, Ling Sun

**Affiliations:** 1 Department of Cardiology, The Affiliated Changzhou No. 2 People’s Hospital of Nanjing Medical University, 29 Xinglong Alley, Changzhou, Jiangsu 213000 China; 2 Graduate School of Dalian Medical University, Dalian Medical University, Dalian, Liaoning, 116000 China

**Keywords:** Acute myocardial infarction, Dapagliflozin, Heart failure, Type 2 diabetes mellitus, Sodium-glucose cotransporter-2 inhibitors

## Abstract

**Objective:**

The aim of this study was to investigate the effect of dapagliflozin (DAPA) on the rate of heart failure rehospitalization in patients with acute myocardial infarction (AMI) and type 2 diabetes mellitus (T2DM).

**Methods:**

AMI patients with T2DM from CZ-AMI registry between January 2017 and January 2021 were enrolled in this study. Patients were stratified into DAPA users and non-DAPA users. The primary outcome was the incidence of heart failure rehospitalization. Kaplan–Meier analysis and Cox regressions were performed to evaluate the prognostic significance of DAPA. Propensity score matching (PSM) was performed to minimize the bias of confounding factors and facilitate the comparability between groups. The enrolled patients were matched with a propensity score of 1:1.

**Results:**

A total of 961 patients were included, and 132 (13.74%) heart failure rehospitalizations occurred during a median follow-up of 540 days. In the Kaplan–Meier analysis, DAPA users had a statistically significantly lower rate of heart failure rehospitalization than non-DAPA users (*p* < 0.0001). Multivariate Cox analysis showed that DAPA was an independent protective factor for heart failure rehospitalization risk after discharge (HR = 0.498, 95% CI = 0.296 ~ 0.831, *p* = 0.001). After 1:1 propensity score matching, survival analysis showed a lower cumulative risk of heart failure rehospitalization in DAPA users than in non-DAPA users (*p* = 0.0007). In-hospital and continued use of DAPA remained significantly associated with a reduced risk of heart failure rehospitalization (HR = 0.417, 95% CI = 0.417 ~ 0.838, *p* = 0.001). Results were consistent across sensitivity and subgroup analyses.

**Conclusion:**

In patients with diabetic AMI, in-hospital and continued use of DAPA after discharge were associated with a significant lower risk of heart failure rehospitalization.

**Supplementary Information:**

The online version contains supplementary material available at 10.1007/s00228-023-03495-3.

## Introduction

Heart failure (HF), affecting approximately 40 million people worldwide, is most likely to be caused by coronary artery atherosclerotic heart disease [1–4]. Over the past 40 years, the prevalence of HF caused by myocardial infarction (MI) has increased by 26% and 48% in men and women, respectively [5]. It is estimated that more than 8 million people aged over 18 will be affected by HF by 2030 [6, 7]. Despite effective treatments, the prognosis for patients with HF remains poor [8]. HF is the leading cause of hospitalization in adults, with a 1-year mortality rate of 10–35% in various cohorts [9–12]. In the past, neurohormonal antagonist drugs (renin-angiotensin system inhibitors, β blockers, and mineralocorticoid receptor antagonists) have laid a cornerstone for the pharmaceutic treatment of HF [13].

Sodium-glucose cotransport protein 2 (SGLT2) inhibitors, originally developed as glucose-lowering agents for the treatment of type 2 diabetes mellitus (T2DM), can decrease the risk of death and other adverse outcomes in patients with chronic HF and reduced ejection fraction (i.e., left ventricular ejection fraction ≤ 40%), or chronic kidney disease, despite the presence of T2DM. Current clinical guidelines strongly recommend the use of SGLT2 inhibitors in patients with chronic HF and reduced ejection fraction [14–16]. The mechanisms by which SGLT2 inhibitors improve HF outcomes are still being investigated, presumably involving regulation on hemodynamics [17, 18], myocardial energy and loading, endothelial function and inflammation, and progression of renal disease [18–21]. The EMPAREG OUTCOMES trial showed that SGLT2 inhibitors improved cardiovascular mortality in MI [22, 23]. However, the long-term outcomes of SGLT2 inhibitors in AMI patients are unclear.

The aim of this study was to investigate the relationship between dapagliflozin (DAPA), a SGLT2 inhibitor, and rehospitalization for HF after MI in patients with AMI combined with T2DM.

## Materials and methods

### Ethics

The study was approved by the institutional ethics committee of the Affiliated Changzhou No. 2 People’s Hospital of Nanjing Medical University (No. 2020-KY253-01). All patients enrolled into the current study provided signed informed consent.

### Study participants

In total, 2291 AMI patients from CZ-AMI Registry (Changzhou AMI Registry, ChiCTR1800014583) between January 2017 and January 2021 were initially included in this study. Briefly, CZ-AMI Registry was a single-center, retrospective, observational cohort study of patients with AMI. The study was conducted in the Department of Cardiology, Changzhou No. 2 People’s Hospital.

Included were those who (1) had T2DM; (2) had undergone coronary angiography and percutaneous coronary intervention treatment during hospitalization; and (3) had an age of ≥ 18 years.

AMI was diagnosed according to the status of myocardial necrosis [24]. The following criteria supported the diagnosis of myocardial infarction: at least one cardiac biomarker value (preferably cardiac troponin [cTnI]) was above the 99th percentile upper reference limit (URL), and at least one of the following: (1) symptoms of ischemia; (2) new or presumed new significant ST-segment T-wave (ST-T) changes or new left bundle branch block (LBBB); (3) development of pathological Q waves in the ECG; (4) new loss of live myocardium or new imaging evidence of localized ventricular wall motion abnormalities; (5) identification of intracoronary thrombus by angiography or autopsy.

T2DM was diagnosed as follows [25]: (1) fasting blood glucose levels on another day ≥ 126 mg/dL; (2) alternatively, typical symptoms and non-fasting blood glucose ≥ 200 mg/dL; (3) a 2-h blood glucose level of 200 mg/dL in an oral glucose tolerance test (OGTT). Therefore, a total of 984 eligible individuals included in the study.

Exclusion criteria included (1) use of other kinds of SGLT2 inhibitors (5 patients used empagliflozin); (2) use of SGLT2 inhibitors before admission (*n* = 3); and (3) unexpected discontinuation of DAPA after discharge for various reasons (3 patients discontinued the drug because of adverse reactions, and 12 patients for other reasons). Finally, a total of 961 patients were included in the current study.

**Fig. 1:**
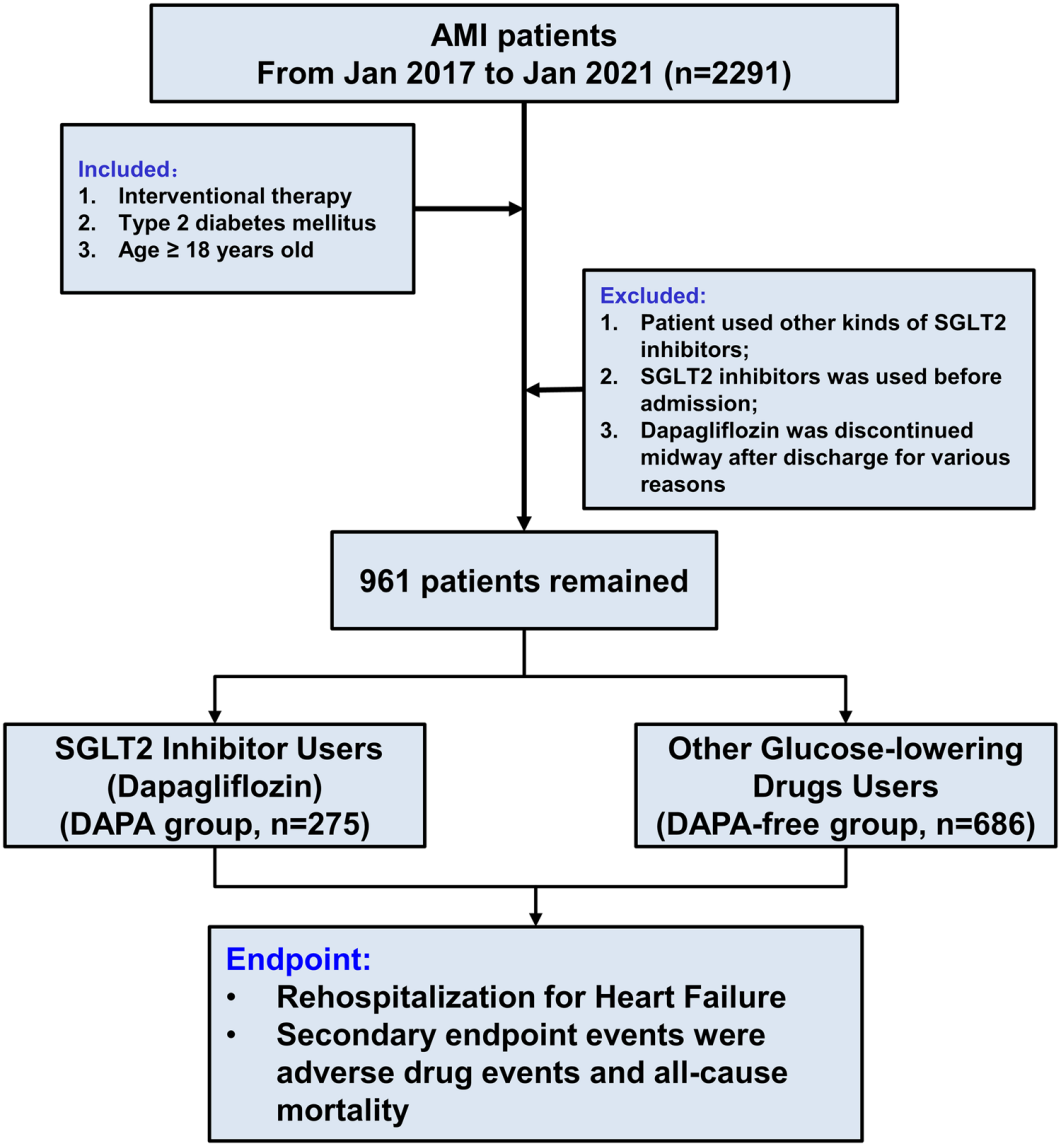
Flow diagram of the selection process of patients

Patients were then stratified into DAPA users (DAPA group) and non-DAPA users (DAPA-Free group). DAPA users received oral DAPA 10 mg (Tablet Forxiga 10 mg, AstraZeneca, Sweden) once daily. Non-DAPA users received other kinds of glucose-lowering drugs (including sulfonylureas, glinides, α-glucosidase inhibitors, dipeptidyl-peptidase IV [DPP-4] inhibitors, insulin). Since all enrolled patients used contrast agents during the procedural, no patients used metformin. The study flowchart is illustrated in Fig. 1.

### Data collections

Using an electronic medical system, we followed up all the patients for general condition, vital signs, procedural-related indicators, medication use, outpatient visits, and rehospitalization. Each patient’s weight and height were collected to calculate body mass index (BMI) by dividing weight (kg) by height squared (m). Systolic blood pressure (SBP) and diastolic blood pressure (DBP) were measured in a calm state at admission. Prior to study procedures, the patient’s fasting blood indices were recorded, including white blood cell (WBC), neutrophil percentage, hemoglobin, blood albumin, blood uric acid, thyroid stimulating hormone (TSH), free triiodothyronine (FT3), free thyroxine (FT4), hemoglobin A1c (HbA1c), and blood glucose levels. For N-terminal pro–brain natriuretic peptide (NT-pro BNP) and troponin-T (TNT), the maximal value was used during hospitalization. From the computerized case system, we gathered information about emergency percutaneous coronary intervention (e-PCI), criminal vascularization, stent placement, and intraoperative hypotension.

### Endpoints

The primary endpoint event was readmission to hospital for HF. The definition of HF was as follows: (1) patients with symptoms and/or signs of HF caused by a structural and/or functional cardiac abnormality and (2) corroborated by at least one of the following: (a) plasma B-type natriuretic peptide (BNP) > 35 pg/mL or N-terminal B-type natriuretic peptide (NT-pro BNP) > 125 pg/mL; (b) evidence of cardiogenic pulmonary or systemic congestion obtained by imaging examination (such as chest radiography and echocardiography) or hemodynamic monitoring (such as right heart catheterization and pulmonary artery catheterization) [26, 27]. Secondary endpoint events were adverse drug events (including volume depletion, hyperkalemia, and ketoacidosis) and all-cause mortality.

### Statistical analyses

The missing variables are shown in Supplementary Table 1. Multiple imputation was used to give each missing variable a value. First, several datasets containing all missing variables were generated. Second, these datasets were used to build several complementary models, usually generalized linear models. Third, these models were integrated together and their performances were evaluated. Finally, the complete dataset was put out [28, 29]. Categorical variables were described using frequencies and percentages, and differences between groups were identified using chi-square tests or Fisher’s exact test. Continuous variables were represented as mean ± standard deviation or mean of median and interquartile range (IQR), and compared using Student’s *t*-test or Mann–Whitney *u*-test. Kaplan–Meier analysis and Cox regressions were performed to evaluate the prognostic significance of DAPA. For survival analysis, “day 0” represented the day that the patient was admitted for AMI. Follow-up time was defined as the time from “day 0” to the occurrence of endpoints (HF rehospitalization or all-cause mortality). Propensity score matching (PSM) was performed to minimize the bias of confounding factors and facilitate the comparability between groups. Age, gender, and Killip heart functional classification were included as matching variables. A 1:1 PSM, with greedy nearest neighbor matching and caliper 0.01, was employed for matching with sex to eliminate bias and compensate for the effect of potential confounders. Standardized mean difference (SMD) was used to compare the baseline characteristics of the two groups.

Statistical analysis was carried out using R software (version 4.1.2). Graphs were created using R software and GraphPad Prism (version 8.3.0). *p* < 0.05 was deemed as statistically different.

## Results

### Baseline characteristics

From January 2017 through January 2021, 2291 patients with AMI admitted to The Affiliated Changzhou No. 2 People’s Hospital of Nanjing Medical University were retrospectively collected into this study initially. Among them, 961 adult AMI patients with T2DM who received interventional therapy were finally included. There were 275 (28.6%) patients in DAPA group and 686 (71.4%) patients in DAPA-Free group. Baseline clinical data between the two groups are shown and compared in Table 1. A more detailed comparison of baseline clinical data between the two groups is provided in Supplementary Table 2.

**Table 1:** Baseline characteristics before and after matching

	**Before matching**			**After matching**		
	**DAPA (*n* = 275)**	**DAPA-free (*n* = 686)**	***P value***	**DAPA (*n* = 231)**	**DAPA-free (*n* = 231)**	***P value***
**Demographic**						
Age (years)	61.97 (13.22)	67.22 (12.15)	< 0.001	63.80 (12.07)	63.80 (12.07)	1
Gender (male)	209 (76.0)	451 (65.7)	0.003	181 (78.4)	181 (78.4)	1
Smoking (%)	115(41.8)	288(42.0)	1	96(41.6)	116(50.2)	0.076
Drinking(%)	32 (11.6)	66 (9.6)	0.415	27 (11.7)	26 (11.3)	1
BMI (kg/m^2^)	25.68 (3.75)	24.40 (3.65)	< 0.001	25.40 (3.33)	24.60 (3.94)	0.019
SBP (mmHg)	133.52 (23.97)	133.24 (25.19)	0.871	134.18 (23.75)	133.54 (24.35)	0.776
DBP (mmHg)	81.44 (14.98)	78.51 (16.06)	0.009	81.14 (14.83)	81.01 (17.42)	0.931
HR (bpm)	82.76 (14.78)	81.22 (16.75)	0.183	80.99 (14.20)	81.26 (16.71)	0.85
STEMI (%)	167 (60.7)	398 (58.0)	0.485	139 (60.2)	142 (61.5)	0.849
NSTEMI (%)	108(39.3)	288(42.0)	0.485	92(39.7)	89(38.5)	0.849
Hypertension (%)	214 (77.8)	538 (78.4)	0.905	186 (80.5)	179 (77.5)	0.493
Killip class ≥ 3 (%)	41 (14.9)	76 (11.1)	0.126	22 (9.5)	22 (9.5)	1
HF rehospitalization (%)	19 (6.9)	113 (16.5)	< 0.001	13 (5.6)	35 (15.2)	0.001
**Laboratory results**						
WBC (10^9^/L)	9.90 [8.04, 12.10]	9.01 [6.96, 11.79]	0.001	9.47 [7.88, 11.85]	9.30 [7.30, 12.02]	0.426
Hemoglobin (g/L)	146.00 [135.00, 158.00]	136.00 [120.25, 148.00]	< 0.001	146.00 [134.00, 157.00]	141.00 [128.00, 152.00]	0.002
HbA1c (%)	8.10 [7.10, 9.50]	7.60 [6.60, 8.78]	< 0.001	8.04 [7.00, 9.50]	7.80 [6.70, 8.85]	0.014
Glucose (mmol/L)	8.78 [7.04, 11.75]	8.57 [6.61, 11.23]	0.178	8.67 [6.90, 11.30]	8.83 [6.69, 11.30]	0.954
Creatinine (μmmol/L)	69.90 [58.80, 83.80]	75.20 [60.50, 96.38]	< 0.001	70.20 [59.95, 83.75]	72.10 [60.65, 92.85]	0.027
NT-proBNP (ng/mL)/500	819.00 [267.50, 2665.00]	1275.00 [258.75, 3065.00]	0.013	753.00 [213.00, 2440.00]	1050.00 [245.50, 3105.00]	0.077
LVEF (%)	49.67 (9.87)	49.86 (9.10)	0.771	50.24 (9.07)	50.41 (9.04)	0.836

Values are mean + SD, *n* (%), or median (inter-quartile range)

*BMI* body mass index, *HR* heart rate, *SBP* systolic blood pressure, *DBP* diastolic blood pressure, *STEMI* ST segment elevation myocardial infarction, *WBC* white blood cell, *HbA1c* hemoglobin A1c, *UA* uric acid, *TSH* thyroid stimulating hormone, *FT3* free triiodothyronine, *FT4* free thyroxine, *TNT* troponin-T, *NT-pro BNP* N-terminal pro–brain natriuretic peptide, *LVEF* left ventricular ejection fraction, *e-PCI* emergency percutaneous coronary intervention, *ACEI/ARB* angiotensin-converting enzyme inhibitors/angiotensin receptor blockers, *GP IIb/IIIa receptor antagonists*, platelet surface glycoprotein IIb/IIIa receptor antagonist

Before matching, the proportion of men was higher in the DAPA group than in the DAPA-Free group (*p* = 0.003), while the mean age was considerably lower in the DAPA group (*p* < 0.001). The proportion of Killip class ≥ 3 showed no difference between the DAPA group and the DAPA-Free group (*p* = 0.126) (Table 1).

### Medication use

Except for the use of ACEI/ARB, there were no significant differences in other medication uses between the two groups before matching (Supplementary Table 2). We additionally analyzed the percentage of patients using ACE/ARBs, beta-blockers, and MRAs in patients with reduced EF (EF ≤ 40%). There were no significant differences between the two groups (Supplementary Table 3). Other glucose-lowering drugs used are listed in in Supplementary Table 4. There was no significant difference between the two groups.

### Follow-up and primary endpoint

The median follow-up time was 540 days. The DAPA group showed a lower HF rehospitalization rate than the DAPA-Free group (6.9% vs. 16.5%; *p* < 0.001; Table 1). In the matched cohort, the HF rehospitalization rate was also lower significantly in the DAPA group than in the DAPA-Free group (5.6% vs. 15.2%; *p* = 0.001; Table 1).

**Fig. 2:**
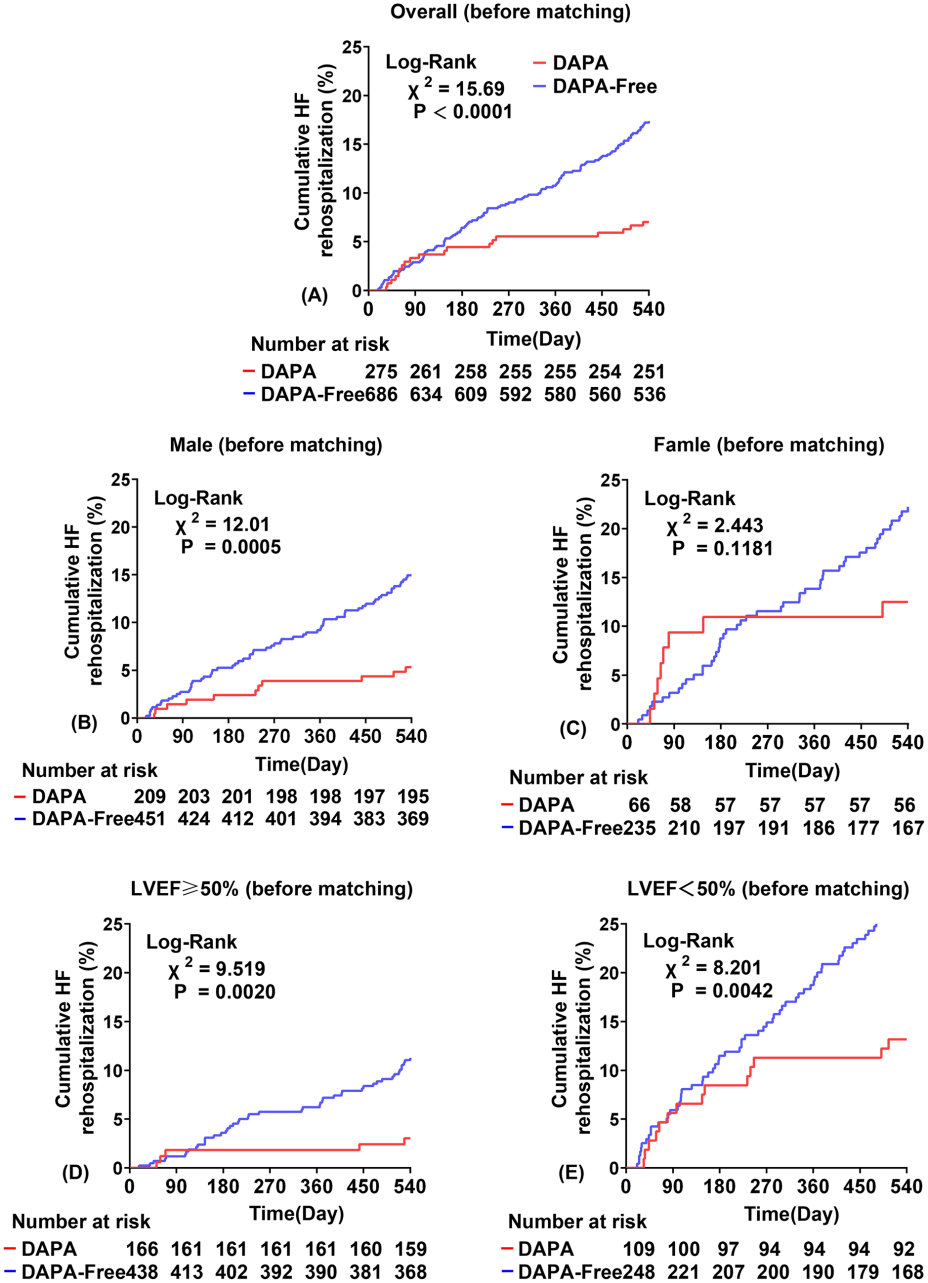
Plot of cumulative heart failure readmission rates stratified by DAPA administration. All lines have the same meaning as the labels in (**A**). Unstratified pressure group before PM (**A**); male subgroup before PM (**B**); female subgroup before PM (**C**); LVEF greater than or equal to 50% before PM (**D**); LVEF less than 50% before PM (**E**)

Kaplan–Meier survival analysis showed a higher rate of HF rehospitalization in the DAPA-Free group than in the DAPA group (log-rank *p* < 0.0001, Fig. 2). In male subgroups, the rate of HF rehospitalization was higher in the DAPA-Free group than in the DAPA group, regardless of whether the ejection fraction was below 50% (log-rank *p* = 0.0005, 0.0020, 0.0042, Fig. 2). However, in the female subgroup, there was no significant difference in the rate of HF rehospitalization between the DAPA and DAPA-Free groups (log-rank *p* = 0.1181, Fig. 2).

Five risk-predicting models were also developed based on the results of Cox regression analysis. In model 1, the use of DAPA reduced the risk of rehospitalization for HF in AMI patients by 61.2% (HR = 0.388, 95% CI: 0.239–0.631). Model 2 was adjusted for age and sex based on model l. Model 3 was adjusted for demographic variables with *p* < 0.05 based on model 2. Model 4 was adjusted for laboratory variables with *p* < 0.05 based on model 3. Model 5 was adjusted for procedural-related variables with *p* < 0.05 based on model 4. Results showed that DAPA reduced the risk of HF rehospitalization in AMI patients (Table 2). Also, a univariate Cox regression analysis was performed for all statistical variables, and significant variables (*p* < 0.05) were included in the Cox multivariate regression analysis (Supplementary Table 5).

**Table 2:** Univariate and multivariable Cox proportional risk analysis of dapagliflozin against heart failure rehospitalization in the overall population

Before matching		Hazard ratio	95% CI	*P-value*
Model 1	DAPA-Free	Reference		
	DAPA	0.388	(0.239 ~ 0.631)	< 0.001
Model 2	DAPA-Free	Reference		
	DAPA	0.488	(0.299 ~ 0.797)	0.004
Model 3	DAPA-Free	Reference		
	DAPA	0.502	(0.307 ~ 0.821)	0.006
Model 4	DAPA-Free	Reference		
	DAPA	0.576	(0.344 ~ 0.963)	0.035
Model 5	DAPA-Free	Reference		
	DAPA	0.498	(0.296 ~ 0.839)	0.008

Model 1: Unadjusted

Model 2: Adjusted for age, sex

Model 3: Model 2 + smoking, drink, BMI, DBP

Model 4: Model 3 + WBC + creatinine + hemoglobin + albumin + HbA1c + FT3 + FT4 + TNI + NT-pro BNP

Model 5: Model 4 + hypertension + KILLIP ≥ 3 + e-PCI + Stent + LAD + ACEI/ARB + LCX + β-blocker

### PSM analysis

**Fig. 3:**
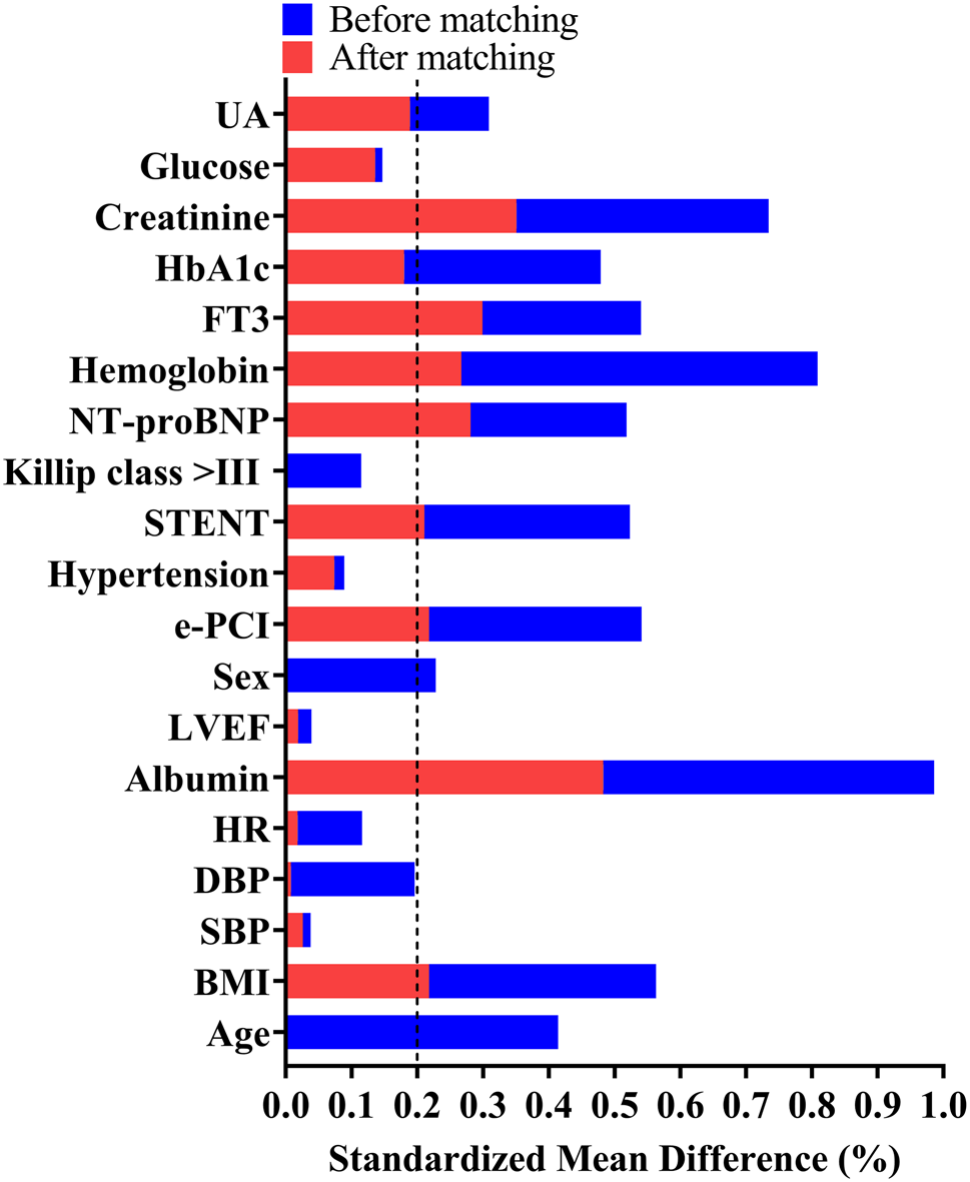
Balance checks of each variable after propensity score matching analysis. Standardized differences of all the variables were illustrated. UA, uric acid; HbA1c, hemoglobin A1c; FT3, free triiodothyronine; NT-pro BNP, N-terminal pro–brain natriuretic peptide; LVEF, left ventricular ejection fraction; HR, heart rate; SBP, systolic blood pressure; DBP, diastolic blood pressure

**Fig. 4:**
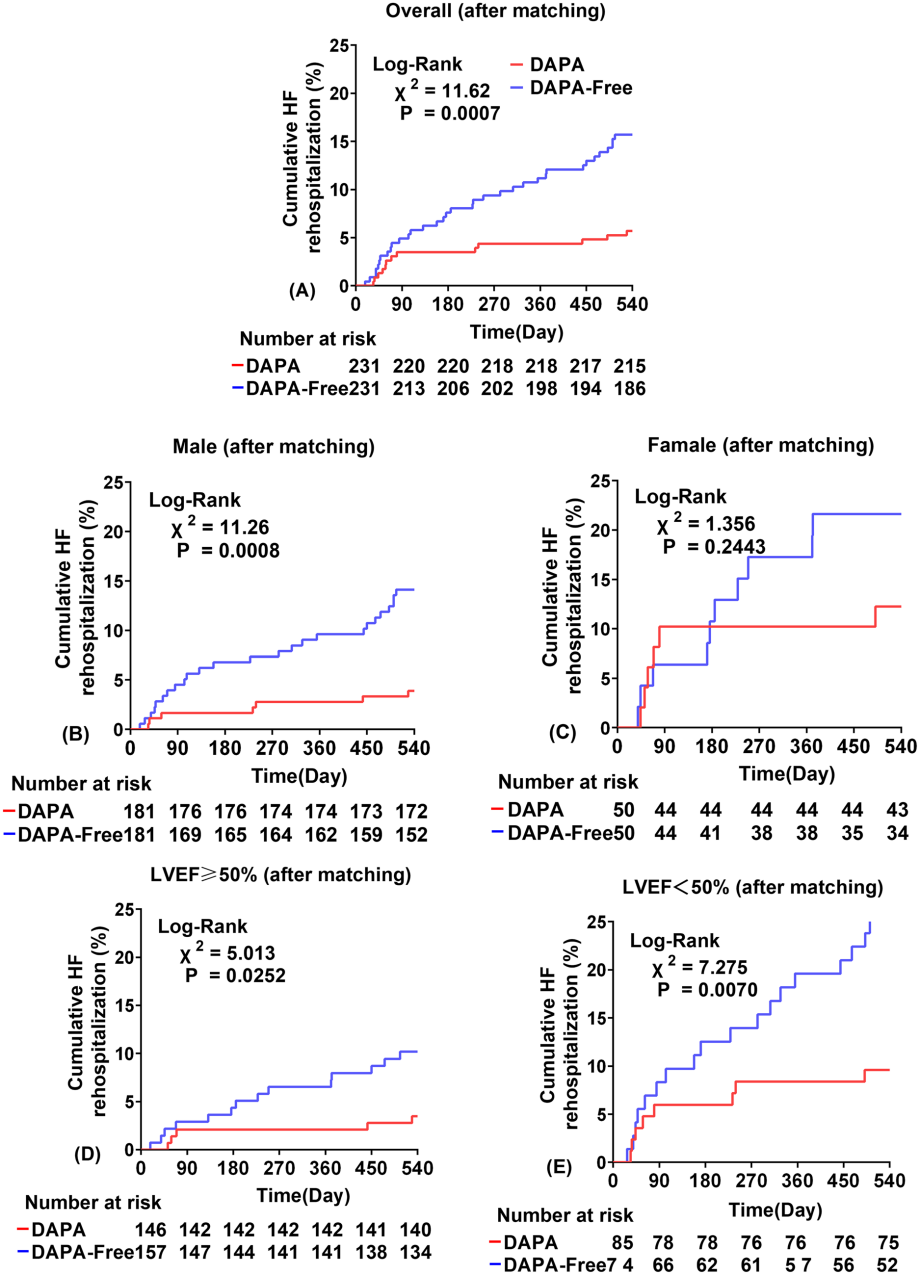
Plot of cumulative heart failure readmission rates stratified by DAPA administration; all lines have the same meaning as the labels in **A**. Unstratified pressure group after PM (**A**); male subgroup after PM (**B**); female subgroup after PM (**C**); LVEF greater than or equal to 50% after PM (**D**); LVEF less than 50% after PM (**E**)

Using a 1:1 PSM, 231 patients taking DAPA were eventually matched with 231 patients taking no DAPA. Some of the observed parameters differed between the DAPA group and the DAPA-Free group after PSM; however, the variation was less significant than that before PSM (Table 1 and Supplementary Table 2). The balance between the groups was assessed (Fig. 3). After matching, the Kaplan–Meier survival analysis showed that the DAPA-Free group had a higher HF rehospitalization rate than the DAPA group (log-rank *p* = 0.0007, Fig. 4). All subgroups, except for the female subgroups, demonstrated a reduction in the rate of HF rehospitalization in AMI patients with DAPA group (log-rank *p* = 0.2443, Fig. 4).

After PSM, series Cox regression analyses were performed and 5 risk-predicting models were created to assess the relationship between DAPA use and the risk of rehospitalization for HF after AMI. In model 1, use of DAPA reduced the risk of HF rehospitalization by 65.3% (HR = 0.347, 95% CI: 0.184–0.657, *p* = 0.001); in model 2 adjusted for age and sex, DAPA reduced the risk of HF rehospitalization by 67.7% (HR = 0.323, 95% CI: 0.171–0.611, *p* < 0.001); in model 3 adjusted for general health status (BMI), DAPA reduced the risk of HF rehospitalization by 68.3% (HR = 0.318, 95% CI: 0.168–0.603, *p* < 0.001); in model 4 adjusted for significant laboratory indicator variables in Table 1, DAPA reduced the risk of rehospitalization for HF by 55.7% (HR = 0.443, 95% CI: 0.221–0.887, *p* = 0.021); in model 5 adjusted for surgical variables in Table 1, DAPA reduced the risk of rehospitalization for HF by 58.3% (HR = 0.417, 95% CI: 0.207–0.838, *p* < 0.001). Also, the univariate Cox regression analysis was performed for all statistical variables after PSM, and all significant variables were absorbed into the multivariate Cox regression analysis (Supplementary Table 6). DAPA significantly reduced the risk of HF rehospitalization in patients with AMI after adjustment (Table 3). At 1 year of follow-up, the DAPA group had significantly higher left ventricular ejection fraction (LVEF) values than the DAPA-free group (*p* = 0.0214, Supplementary Fig. 1).

**Table 3:** Univariate and multivariate Cox proportional risk analysis of dapagliflozin and heart failure rehospitalization in a matched AMI cohort

After matching		Hazard ratio	95%CI	*P-value*
Model 1	DAPA-Free	Reference		
	DAPA	0.347	(0.184 ~ 0.657)	0.001
Model 2	DAPA-Free	Reference		
	DAPA	0.323	(0.171 ~ 0.611)	< 0.001
Model 3	DAPA-Free	Reference		
	DAPA	0.318	(0.168 ~ 0.603)	< 0.001
Model 4	DAPA-Free	Reference		
	DAPA	0.443	(0.221 ~ 0.887)	0.021
Model 5	DAPA-Free	Reference		
	DAPA	0.417	(0.207 ~ 0.838)	0.001

Model 1: Unadjusted

Model 2: Adjusted for age, sex

Model 3: Model 2 + BMI

Model 4: Model 3 + hemoglobin + albumin + HbA1c + creatinine + FT3 + FT4 + TNI + NT-pro BNP

Model 5: Model 4 + e-PCI + Stent + LAD + ACEI/ARB

### Secondary endpoints

DAPA-related adverse events were also evaluated in the entire cohort. Throughout the follow-up period, ketoacidosis occurred in 1 patient and volume depletion in 2 patients (these 3 patients discontinued DAPA and were excluded from the further analysis). Hyperkalemia was not observed in all patients using DAPA. Kaplan–Meier survival analysis also showed that all-cause mortality was higher in the DAPA-Free group than in the DAPA group in both entire and matched cohorts (Log-rank *p* < 0.05, Supplementary Fig. 2).

### Subgroup analysis

**Fig. 5:**
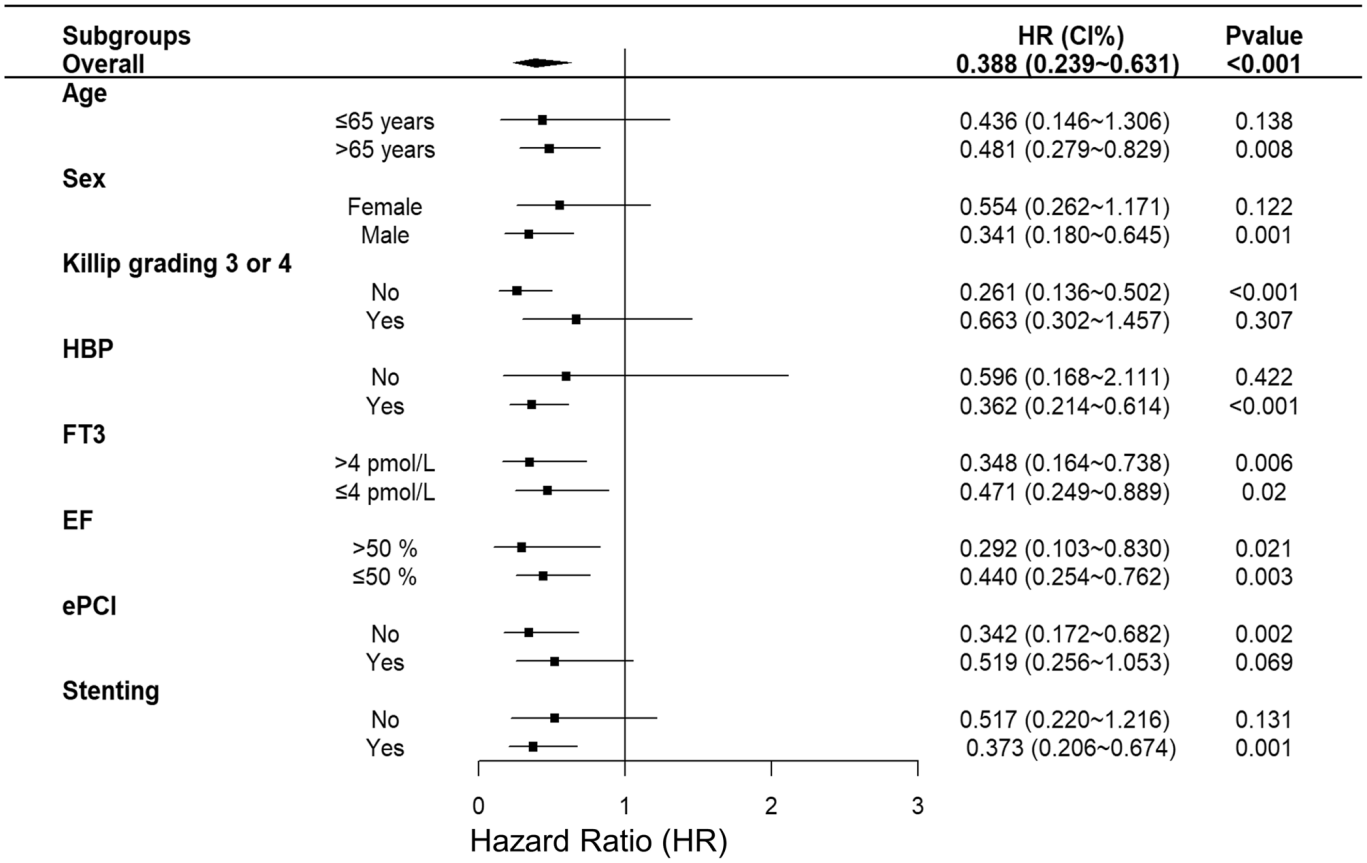
Subgroup analysis forest plots

Subgroup analysis was performed by age, sex, Killip classification, hypertension, FT3, LVEF, e-PCI, and Stent. The association between DAPA and rehospitalization for HF in various subgroups was further investigated. Subgroup analysis forest plots showed that DAPA could reduce the risk of rehospitalization for HF (Fig. 5).

## Discussion

In the current study, we found that in-hospital and continued use of DAPA after discharge was associated with a significant lower risk of HF rehospitalization in patients with diabetic AMI, compared with non-DAPA users. Other independent predictors of the HF rehospitalization included age, hypertension, uric acid, and Killip class. Patients with AMI and T2DM were more likely to benefit from in-hospital and continued use of DAPA. However, prospective clinical trials with a larger sample size and a longer follow-up are still required.

During the follow-up period, a total of 150 patients were hospitalized for heart failure, with a rate of 15%. It is reported in a retrospective study with a large sample of 77,363 AMI patients [30] that the heart failure rehospitalization rate was about 22% during a 5-year follow-up. The difference in this rate may be attributed to the smaller sample size in our study, as well as the geographical, dietary, climatic factors, and patients’ compliance.

We found no significant difference in the risk of HF rehospitalization between female subgroups. There are two main possible reasons. Firstly, the prevalence of AMI is higher in male than in female patients [31]. In our study, there were 66 female patients in DAPA group. The sample size is relatively small, and therefore may contribute to the lack of significant differences in the subgroup of females. Secondly, female patients have a worse prognosis post AMI [30]. During the follow-up, some female patients may have died of various reasons and could not achieve the primary endpoint. This may have also influenced the results of subgroup analysis.

In this study, we also found that most deaths occurred within 30 days post AMI and the difference in all-cause mortality was more evident in the early phase of follow-up. In another study, AMI patients undergoing percutaneous coronary intervention (PCI) and complicated with cardiogenic shock were enrolled and analyzed. Most deaths also occurred within 30 days after AMI, which is consistent with our results [32]. In the early phase post AMI, due to severe myocardial injury, cardiac dysfunction may lead to heart rupture, or papillary muscle dysfunction or rupture. These damages may end up with HF and even sudden death. In addition, various ventricular arrhythmias may appear in the early stage after heart injury, which can also result in sudden death [33]. Therefore, patients with AMI are more likely to have serious complications in the early phase after AMI. However, this study also indicated that early use of DAPA can reduce the risk of death in AMI patients more significantly.

Recurrent myocardial infarction (MI) is often followed by chronic HF, malignant arrhythmias, and cardiovascular death [34]. SGLT2 inhibitors, a novel oral hypoglycemic agent, have shown in recent clinical studies to significantly reduce the incidence of composite cardiovascular death or worsening HF events in HF patients with mildly reduced or preserved ejection fraction over a median follow-up of 2.0–2.5 years [35, 36]. However, whether SGLT2 inhibitors are effective in the early post-infarction period lacks evidence. Furthermore, considering that patients with a long history of AMI are more likely to have shared indications for SGLT2 inhibitors (e.g., T2DM or HF), it may be hasty to determine that the benefit of SGLT2 inhibitors for patients is in the context of AMI. Despite their proximate therapeutic spectra, trials in AMI populations are needed to confirm whether their treatment effects are consistent.

Existing studies suggest that in patients with T2DM and atherosclerotic cardiovascular disease, SGLT2 inhibitors reduce cardiovascular all-cause mortality and death from renal disorders, renal replacement therapy, or doubling of the serum creatinine level [37]. In contrast, this study focused on the ability of SGLT2 inhibitor in predicting rehospitalization for HF after AMI in patients combined with T2DM. One strength of this study was the introduction of propensity score matching, which allows direct comparison between the treatment and control groups, as a randomized controlled trial does [38], as well as robust error specification of the PS model [39]. In conclusion, more clinical evidence is needed in the future to confirm whether SGLT2 inhibitors should be used earlier in AMI patients.

The incidence of adverse events in this study was not high. On the one hand, this is related to the small sample size; on the other hand, our patients were all diabetic and they regularly visited a clinic for glycemic assessment and health education, which may lower the incidence of adverse events. However, the safety of SGLT2 inhibitors still requires the verification with more clinical data.

The formation of fibrous scar tissue and ventricular remodeling after AMI, along with a progressive decrease in myocardial contractility and ultimately heart failure, have been associated with death. Renal injury in the early stages of AMI has been associated with a poor prognosis in the short term and can increase long-term mortality [40]. The development of malignant arrhythmias is an important cause of early death in AMI patients [33]. Early identification of patients at a high risk of various complications is important, and an artificial intelligence model for predicting acute kidney injury risk and a new scoring system for predicting ventricular arrhythmia risk have been developed [33, 41]. In our previous studies, we have also found that a low level of free triiodothyronine is independently associated with the short-term outcomes in patients with AMI [42]. Prevention is important, but treatment is equally vital and SGLT2 inhibitors provide a new therapeutic direction for the prognosis of AMI patients. SGLT2 inhibitors may reduce the risk of acute myocardial infarction via mechanisms responsible for attenuating neurohormonal activation, cardiomyocyte necrosis, and reperfusion injury. It may also facilitate coronary blood flow and reduce ventricular load by enhancing endothelial function and vasodilation, improving myocardial energy metabolism and contractility, and other mechanisms [43–46]. With the reversal of cardiac enlargement, rhythm abnormalities, and myocardial fibrosis, HF is finally cured [47, 48]. In addition, outside the heart, SGLT2 inhibitors may also indirectly protect the cardiorenal axis by reducing intra-glomerular pressure and increasing erythropoietin production, among many other mechanisms [49, 50].

## Limitations

There are several limitations in the current study. First, this study is a single-center retrospective study with a small sample size. The randomized controlled trials with larger-size samples and longer follow-up are still required in the future. Second, the patients in the DAPA-Free group may also use several kinds of glucose-lowering drugs, the effects of which were not assessed separately. Third, the current study mainly observed effects of DAPA; the efficacy and safety of other SGLT2 inhibitors, such as canagliflozin and empagliflozin, in patients with AMI should also be evaluated. Finally, patients discontinuing DAPA use were excluded from the analysis. In fact, this discontinuation may be due to the incidence of adverse events. This might have resulted in a lower observed incidence of adverse events.

## Conclusions

In patients with diabetic AMI, in-hospital and continued use of DAPA after discharge from hospital were associated with a significant lower risk of HF rehospitalization.

## Supplementary Information

Below is the link to the electronic supplementary material.

Supplementary file1 (DOCX 24 KB)

Supplementary file2 (DOCX 42 KB)

## Data Availability

The datasets and materials used in the study are available from the corresponding author.
