# Supplementary material for: Dapagliflozin reduces risk of heart failure rehospitalization in diabetic acute myocardial infarction patients: a propensity score-matched analysis
Source: Eur J Clin Pharmacol. 2023 Apr 26;79(7):915–26. doi: 10.1007/s00228-023-03495-3 (PMC10276777; doi:10.1007/s00228-023-03495-3)
Supplement: Supplementary file 1 — Supplementary file1 (DOCX 24 KB) [file 228_2023_3495_MOESM1_ESM.docx]

**Supplemntary Figure 1.** Left ventricular ejection fraction values at one-year after myocardial infarction between DAPA group and DAPA-Free group. N=180 in DAPA group, n=105 in DAPA-Free group. Data are presented as 48.6 ± 8.2(%). Statistical analysis was performed with student t test. *P < 0.05.

**Supplemntary Figure 2.** Survival analysis between DAPA group and DAPA-Free group before **(A)** and after propensity score matching **(B)**.
