# Supplementary material for: Dapagliflozin reduces risk of heart failure rehospitalization in diabetic acute myocardial infarction patients: a propensity score-matched analysis
Source: Eur J Clin Pharmacol. 2023 Apr 26;79(7):915–26. doi: 10.1007/s00228-023-03495-3 (PMC10276777; doi:10.1007/s00228-023-03495-3)
Supplement: Supplementary file 2 — Supplementary file2 (DOCX 42 KB) [file 228_2023_3495_MOESM2_ESM.docx]

**Supplementary Table 1. Missing rate of each variable**

|  | **Collected cases** | **Missing cases** | **Missing rate (%)** |
| --- | --- | --- | --- |
| e-PCI | 961 | 0 | .0 |
| Age | 961 | 0 | 0 |
| Gender | 961 | 0 | 0 |
| Heart rate | 961 | 0 | 0 |
| Smoking | 961 | 0 | .0 |
| Drinking | 961 | 0 | .0 |
| Hypertension | 961 | 0 | .0 |
| Hight | 939 | 22 | 2.3 |
| Weight | 939 | 22 | 2.3 |
| BMI | 939 | 22 | 2.3 |
| SBP | 961 | 0 | .0 |
| DBP | 961 | 0 | .0 |
| WBC | 961 | 0 | .0 |
| Percentage of Neutrophils | 961 | 0 | .0 |
| Hemoglobin | 961 | 0 | .0 |
| Creatinine | 961 | 0 | .0 |
| Glucose | 960 | 1 | .1 |
| Albumin | 935 | 26 | 2.7 |
| UA | 961 | 0 | .0 |
| HbA1C | 937 | 24 | 2.5 |
| TSH | 948 | 13 | 1.4 |
| FT3 | 949 | 12 | 1.2 |
| FT4 | 949 | 12 | 1.2 |
| LVEF | 909 | 52 | 5.4 |
| TNT | 878 | 83 | 8.6 |
| NT-pro BNP | 883 | 78 | 8.1 |
| LM | 961 | 0 | .0 |
| LAD | 961 | 0 | .0 |
| RCA | 961 | 0 | .0 |
| LCX | 961 | 0 | .0 |
| Stent |  | 0 | .0 |
| STEMI | 961 | 0 | .0 |
| Killip class | 961 | 0 | .0 |
| ACEI/ARB | 961 | 0 | .0 |
| β-blocker | 961 | 0 | .0 |
| GP IIb/IIIa receptor antagonists | 961 | 0 | .0 |
| dapagliflozin | 961 | 0 | .0 |

e-PCI: emergency percutaneous coronary intervention; SBP, systolic blood pressure; DBP, diastolic blood pressure;BMI: body mass index; WBC, white blood cell; UA, Uric acid; HbA1c, Hemoglobin A1c; TSH, Thyroid stimulating hormone; FT3, free triiodothyronine; FT4, free thyroxine; LVEF, Left ventricular ejection fraction; NT-pro BNP, N-terminal pro–brain natriuretic peptide; NT-pro BNP, N-terminal pro–brain natriuretic peptide; LM, left main coronary artery; LAD, left anterior descending, RCA, right coronary artery, LCX, left circumflex branch STEMI,ST segment elevation myocardial infarction; ACEI/ARB: Angiotensin-converting enzyme inhibitors/ angiotensin receptor blockers;

**Supplementary Table 2. Baseline Characteristics before and after matching**

|  | **Before matching** | | |  | **After matching** | | |
| --- | --- | --- | --- | --- | --- | --- | --- |
|  | **DAPA**  **(n=275)** | **DAPA-Free**  **(n = 686)** | ***P value*** |  | **DAPA**  **(n=231)** | **DAPA-Free**  **(n = 231)** | ***P value*** |
| **Demographic** |  |  |  |  |  |  |  |
| Age (years) | 61.97 (13.22) | 67.22 (12.15) | <0.001 |  | 63.80 (12.07) | 63.80 (12.07) | 1 |
| Gender (Male) | 209 (76.0) | 451 (65.7) | 0.003 |  | 181 (78.4) | 181 (78.4) | 1 |
| Smoking (%) | 115(41.8) | 288(42.0) | 1 |  | 96(41.6) | 116(50.2) | 0.076 |
| Drinking(%) | 32 (11.6) | 66 (9.6) | 0.415 |  | 27 (11.7) | 26 (11.3) | 1 |
| Hight (cm) | 167.30 (7.16) | 165.31 (7.52) | <0.001 |  | 167.32 (7.17) | 166.84 (7.30) | 0.48 |
| Weight (Kg) | 72.24 (13.71) | 66.90 (11.98) | <0.001 |  | 71.43 (12.36) | 68.73 (13.16) | 0.023 |
| BMI (Kg/m²) | 25.68 (3.75) | 24.40 (3.65) | <0.001 |  | 25.40 (3.33) | 24.60 (3.94) | 0.019 |
| SBP (mmHg) | 133.52 (23.97) | 133.24 (25.19) | 0.871 |  | 134.18 (23.75) | 133.54 (24.35) | 0.776 |
| DBP (mmHg) | 81.44 (14.98) | 78.51 (16.06) | 0.009 |  | 81.14 (14.83) | 81.01 (17.42) | 0.931 |
| HR (bpm) | 82.76 (14.78) | 81.22 (16.75) | 0.183 |  | 80.99 (14.20) | 81.26 (16.71) | 0.85 |
| STEMI (%) | 167 (60.7) | 398 (58.0) | 0.485 |  | 139 (60.2) | 142 (61.5) | 0.849 |
| NSTEMI (%) | 108 (39.3) | 288 (42.0) | 0.485 |  | 92 (39.8) | 89 (38.5) | 0.849 |
| Hypertension (%) | 214 (77.8) | 538 (78.4) | 0.905 |  | 186 (80.5) | 179 (77.5) | 0.493 |
| Killip class ≥ 3 (%) | 41 (14.9) | 76 (11.1) | 0.126 |  | 22 (9.5) | 22 (9.5) | 1 |
| HF- rehospitalization (%) | 19 (6.9) | 113 (16.5) | <0.001 |  | 13 (5.6) | 35 (15.2) | 0.001 |
| **Laboratory results** |  |  |  |  |  |  |  |
| WBC (10⁹/L) | 9.90 [8.04, 12.10] | 9.01 [6.96, 11.79] | 0.001 |  | 9.47 [7.88, 11.85] | 9.30 [7.30, 12.02] | 0.426 |
| Percentage of Neutrophils (%) | 71.93 (12.20) | 74.24 (11.67) | 0.006 |  | 71.11 (12.23) | 74.08 (11.94) | 0.008 |
| Hemoglobin (g/L) | 146.00 [135.00, 158.00] | 136.00 [120.25, 148.00] | <0.001 |  | 146.00 [134.00, 157.00] | 141.00 [128.00, 152.00] | 0.002 |
| Albumin (g/L) | 40.13 (3.61) | 38.18 (4.15) | <0.001 |  | 40.15 (3.46) | 38.40 (3.79) | <0.001 |
| HbA1c (%) | 8.10 [7.10, 9.50] | 7.60 [6.60, 8.78] | <0.001 |  | 8.04 [7.00, 9.50] | 7.80 [6.70, 8.85] | 0.014 |
| Glucose (mmol/L) | 8.78 [7.04, 11.75] | 8.57 [6.61, 11.23] | 0.178 |  | 8.67 [6.90, 11.30] | 8.83 [6.69, 11.30] | 0.954 |
| Creatinine (μmmol/L) | 69.90 [58.80, 83.80] | 75.20 [60.50, 96.38] | <0.001 |  | 70.20 [59.95, 83.75] | 72.10 [60.65, 92.85] | 0.027 |
| UA (μmmol/L) | 318.50 [258.50, 394.50] | 327.95 [268.25, 405.00] | 0.183 |  | 316.00 [256.50, 382.50] | 323.00 [269.50, 400.60] | 0.204 |
| TSH (µIU/mL) | 1.14 [0.77, 1.89] | 1.13 [0.66, 1.99] | 0.917 |  | 1.14 [0.80, 1.84] | 1.00 [0.62, 1.88] | 0.172 |
| FT3 (pmol/L) | 4.20 [3.70, 4.70] | 3.90 [3.40, 4.40] | <0.001 |  | 4.20 [3.70, 4.70] | 4.00 [3.50, 4.46] | 0.002 |
| FT4 (pmol/L) | 16.70 [14.61, 18.66] | 15.60 [13.90, 17.40] | <0.001 |  | 16.50 [14.60, 18.50] | 15.50 [14.05, 16.95] | <0.001 |
| TNT (ng/mL) | 8.68 [2.26, 32.65] | 3.39 [0.97, 17.67] | <0.001 |  | 7.33 [1.96, 31.45] | 4.00 [1.33, 17.65] | 0.009 |
| NT-proBNP (ng/mL)/500 | 819.00 [267.50, 2665.00] | 1275.00 [258.75, 3065.00] | 0.013 |  | 753.00 [213.00, 2440.00] | 1050.00 [245.50, 3105.00] | 0.077 |
| LVEF (%) | 49.67 (9.87) | 49.86 (9.10) | 0.771 |  | 50.24 (9.07) | 50.41 (9.04) | 0.836 |
| **Procedural features** |  |  |  |  |  |  |  |
| e-PCI **(%)** | 152 (55.3) | 270 (39.4) | <0.001 |  | 127 ( 55.0) | 102 (44.2) | 0.026 |
| Left main coronary artery **(%)** | 0 (0) | 6 (0.9) | 0.27 |  | 0 (0) | 3 (1.3) | 0.247 |
| Left anterior descending artery **(%)** | 129 (46.9) | 224 (32.7) | <0.001 |  | 106 (45.9) | 79 (34.2) | 0.014 |
| Right coronary artery **(%)** | 71 (25.8) | 174 (25.4) | 0.949 |  | 60 (26.0) | 63 (27.3) | 0.833 |
| Left circumflex artery **(%)** | 50 (18.2) | 85 (12.4) | 0.026 |  | 40 (17.3) | 27 (11.7) | 0.113 |
| Stent **(%)** | 224 (81.5) | 467 (68.1) | <0.001 |  | 184 (79.7) | 163 (70.6) | 0.031 |
| Low blood pressure **(%)** | 17 (6.2) | 27 (3.9) | 0.182 |  | 15 (6.5) | 7 (3.0) | 0.126 |
| **Medication, n (%)** |  |  |  |  |  |  |  |
| ACEI/ARB **(%)** | 204 (74.2) | 438 (63.8) | 0.003 |  | 172 (74.5) | 148 (64.1) | 0.02 |
| β-blocker **(%)** | 199 (72.4) | 447 (65.2) | 0.038 |  | 169 (73.2) | 154 (66.7) | 0.156 |
| GP IIb/IIIa receptor antagonists **(%)** | 129 (46.9) | 348 (50.7) | 0.318 |  | 104 (45.0) | 122 (52.8) | 0.114 |
| MRAs (%) | 79 (28.7) | 179 (26.1) | 0.452 |  | 64 (27.7) | 63 (27.3) | 1.000 |
| Diuretics (%) | 84 (30.5) | 217 (31.6) | 0.801 |  | 67 (29.0) | 72 (31.2) | 0.685 |

Values are mean + SD, n (%), or median (IOR).

BMI: body mass index; HR: heart rate; SBP, systolic blood pressure; DBP, diastolic blood pressure; STEMI,ST segment elevation myocardial infarction; WBC, white blood cell; HbA1c, Hemoglobin A1c; UA, Uric acid; TSH, Thyroid stimulating hormone; FT3, free triiodothyronine; FT4, free thyroxine; TNT, Troponin-T; NT-pro BNP, N-terminal pro–brain natriuretic peptide; LVEF, Left ventricular ejection fraction; e-PCI: emergency percutaneous coronary intervention; ACEI/ARB: Angiotensin-converting enzyme inhibitors/ angiotensin receptor blockers; GP IIb/IIIa receptor antagonists: Platelet surface glycoprotein IIb/IIIa receptor antagonist. MRAs: mineralocorticoid receptor antagonists; HF: heart failure.

**Supplementary Table 3. Patients with left ventricular ejection fraction less than or equal to 40% regarding the use of ACE/ARBs, β-blockers and MRAs**

|  | DAPA (n=57) | DAPA-Free (n=115) | p-value |
| --- | --- | --- | --- |
| MRAs (%) | 37 (64.9) | 71 (61.7) | 0.812 |
| β-blocker (%) | 40 (70.2) | 83 (72.2) | 0.925 |
| ACEI/ARB (%) | 45 (78.9) | 74 (64.3) | 0.076 |

**Supplementary Table 4. The rate of use of diabetes medications**

|  | DAPA (n=275) | DAPA-Free (n=686) | p-value |
| --- | --- | --- | --- |
| Insulin (%) | 90 (32.7) | 243 (35.4) | 0.472 |
| DPP4i (%) | 23 (8.4) | 38 (5.5) | 0.140 |
| sulfonylureas (%) | 51 (18.5) | 154 (22.4) | 0.212 |
| Glinides (%) | 55 (20.0) | 167(24.3) | 0.149 |
| Alpha-glycosidase inhibitors (%) | 74 (26.9) | 205 (29.9) | 0.401 |

DPP4i, Dipeptidyl peptidase IV inhibitors;

**Supplementary Table 5. Propensity score matching before Cox multifactor regression results**

| **Characteristics** | **Hazard Ratio (95%CI)** | ***P-value*** |
| --- | --- | --- |
| e-PCI | 1.063 (0.698-1.617) | 0.7760 |
| Age | 1.458 (1.206-1.762) | <0.001 |
| Sex | 0.988 (0.659-1.482) | 0.9540 |
| Hypertension | 2.119 (1.21-3.71) | 0.0090 |
| BMI | 0.987 (0.939-1.037) | 0.6020 |
| HR | 1.078 (0.974-1.193) | 0.1480 |
| Hemoglobin | 0.947 (0.856-1.049) | 0.2980 |
| Creatinine | 1.023 (0.978-1.071) | 0.3250 |
| Glucose | 1.03 (0.993-1.069) | 0.1090 |
| Albumin | 0.968 (0.922-1.017) | 0.2030 |
| UA | 1.09 (1.01-1.177) | 0.0260 |
| FT3 | 0.982 (0.837-1.152) | 0.8200 |
| LVEF | 0.954 (0.937-0.971) | <0.001 |
| NT-pro BNP | 1 (0.969-1.031) | 0.9780 |
| Stent | 0.902 (0.6-1.355) | 0.6190 |
| Killip classification≥3 | 1.867 (1.166-2.988) | 0.0090 |
| DAPA | 0.496 (0.295-0.833) | 0.0080 |

**Supplementary Table 6. Propensity score matching after Cox multifactor regression results**

| **Characteristics** | **Hazard Ratio (95%CI)** | **P-value** |
| --- | --- | --- |
| e-PCI | 1.204 (0.58-2.499) | 0.6180 |
| Age | 1.986 (1.373-2.873) | <0.001 |
| Sex | 1.78 (0.86-3.682) | 0.1200 |
| DBP | 0.864 (0.703-1.061) | 0.1630 |
| HR | 1.086 (0.9-1.311) | 0.3890 |
| Hemoglobin | 0.971 (0.815-1.158) | 0.7470 |
| Creatinine | 1.002 (0.915-1.097) | 0.9720 |
| Glucose | 1.002 (0.94-1.068) | 0.9540 |
| Albumin | 0.867 (0.785-0.958) | 0.0050 |
| UA | 1.126 (0.994-1.276) | 0.0620 |
| FT3 | 0.96 (0.643-1.431) | 0.8400 |
| LVEF | 0.944 (0.916-0.972) | <0.001 |
| NT-pro BNP | 0.974 (0.908-1.044) | 0.4560 |
| Left anterior descending artery | 1.011 (0.457-2.238) | 0.9790 |
| Stent | 0.554 (0.254-1.21) | 0.1380 |
| Killip classification≥3 | 2.482 (1.112-5.537) | 0.0260 |
| DAPA | 0.38 (0.186-0.775) | 0.0080 |
